# Female genital self-image and psychological distress: the serial mediating effects of existential shame and limited access to emotion regulation strategies

**DOI:** 10.1007/s00737-025-01662-2

**Published:** 2026-02-04

**Authors:** Isabella Magdala, Magdalena Sánchez-Fernández, Jose M. Mestre

**Affiliations:** 1 Department of Psychology, University of Cadiz (Universidad de Cádiz), Av. República Saharaui, s/n, 11009 Cadiz, Spain; 2 Department of Psychology, Valencian International University, Valencia, Spain

**Keywords:** Female genital self-image, Existential shame, Emotional dysregulation, Psychological distress, Women’s health

## Abstract

**Purpose:**

Concern about female genital self-image (FGSI) is increasing due to its implications for women's well-being. Previous research has linked negative FGSI to psychological distress. However, the underlying mechanisms of this association remain unclear, with shame and emotional dysregulation being potential mediators. This study aimed to test a serial mediation model in which FGSI is associated with psychological distress through shame and emotional dysregulation.

**Methods:**

A cross-sectional design was employed, with a total of 445 women (Age: M = 40.22, SD = 10.69, range = 17–70) completing an online survey.

**Results:**

Linear regression analysis revealed a significant association between psychological distress and both existential shame and limited access to emotion regulation strategies. FGSI and the remaining dimensions of shame and emotional dysregulation did not show a direct significant association with psychological distress. Mediation analysis confirmed that the relationship between FGSI and psychological distress was fully mediated by existential shame, limited access to emotion regulation strategies, and the serial mediation of these two variables.

**Conclusion:**

These findings have important practical implications for the development of preventive and intervention strategies aimed at women with low FGSI, focusing on addressing feelings of maladaptive shame and enhancing effective emotion regulation strategies.

## Highlights

Negative female genital self-image (FGSI) was indirectly associated with psychological distress.

This association was fully mediated by existential shame and difficulties in emotion regulation.

Findings highlight the need for interventions targeting genital self-image, shame, and emotional coping in women.

## Introduction

Female genital self-image (FGSI) is a fundamental aspect of body image that has received increasing attention in psychological research due to its implications for women’s well-being (Alavi-Arjas et al. 2023). FGSI encompasses women’s perceptions, attitudes, and feelings toward their genitalia, including their appearance, odor, and functionality (Herbenick et al. 2011; Herbenick and Reece 2010).

Negative FGSI has been consistently linked to psychological distress. For instance, a positive genital self-image has been found to correlate negatively with sexual distress and depression (Berman et al. 2003). Similarly, FGSI has been associated with sexual distress in premenopausal women with dyspareunia (Pazmany et al. 2013), as well as with the severity of depression and anxiety in pregnant women (Keramat et al. 2021). Additionally, FGSI has been linked to sexual satisfaction and stress in women after vaginal delivery (Sönmez et al. 2024). Despite the growing recognition of its importance, the mechanisms underlying the relationship between FGSI and psychological distress remain insufficiently explored.

One potential explanatory factor in this relationship is shame, a self-conscious moral emotion characterized by feelings of inadequacy and unworthiness (Tangney et al. 2007). In the context of FGSI, shame may stem from internalized societal ideals of female genital appearance, leading to genital dissatisfaction, negative self-perceptions, and increased psychological distress when these ideals are not met (McDougall 2013; Michala 2020). Indeed, research suggests that a negative genital self-image is associated with perceiving menstruation as shameful, indicating a broader connection between how women view their genitalia and their experience of shame (Kvalem et al. 2024).

Scheel et al. (2013) have elucidated the construct of shame by distinguishing its three facets. Bodily shame pertains to feelings of shame associated with physical ideals, appearances, intimacy, and sexuality. This form of shame is typically elicited when an individual’s body standards are transgressed or when parts of their body are exposed in a manner perceived as inappropriate. Cognitive shame encompasses the experience of shame related to one’s cognitive and moral standards, competence, and social standing, and is provoked when an individual perceives a violation of their own social or moral norms. Lastly, existential shame represents a maladaptive dimension characterized by a profound sense of being fundamentally deficient, worthless, or irrelevant as a person. Unlike other forms of shame, existential shame is not confined to a specific situation or behavior but is an intense and pervasive experience of self-directed shame (Scheel et al. 2020).

Shame, in turn, is strongly associated with psychological distress. Studies have shown that shame is a significant risk factor for psychopathology, including depression and anxiety, with external shame—the perception of being judged negatively by others—being particularly linked to these conditions (Callow et al. 2021; DeCou et al. 2021; Velotti et al. 2017). In specific populations, such as transgender individuals, experiences of shame contribute to heightened psychological distress (Carvalho et al. 2022). Similarly, among bereaved adults, shame is a key predictor of complicated grief and depression (LeBlanc et al. 2019). Moreover, shame has been moderately associated with PTSD symptoms, playing a crucial role in trauma-related distress (López-Castro et al. 2019). All these findings suggest that shame may act as a mediator in the relationship between FGSI and psychological distress, warranting further investigation into its specific role in this dynamic.

Additionally, difficulties in emotion regulation may play a critical role in exacerbating psychological distress. Emotional dysregulation is a transdiagnostic risk factor that increases vulnerability to psychopathology (Aldao et al. 2016). Consequently, women with a negative FGSI and difficulties in regulating emotions may struggle to manage feelings of shame and negative self-perceptions, further intensifying their psychological distress. Indeed, prior research has found that maladaptive emotion regulation strategies mediate the relationship between body image dimensions and non-suicidal self-injury in young adults (Duggan et al. 2013). Furthermore, emotional dysregulation has been shown to mediate the link between shame and psychological distress (Remondi et al. 2023; Velotti et al. 2017).

Understanding how shame and emotion dysregulation mediate the relationship between FGSI and psychological distress is crucial for developing more effective psychological interventions. While previous research has independently examined the effects of FGSI, shame, and emotion regulation on mental health, few studies have explored these variables within a serial mediation model.

### The present study

**Fig. 1:**
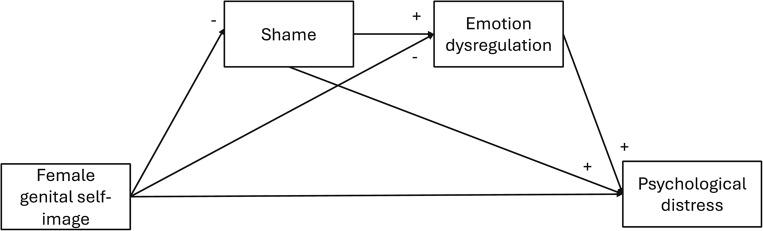
Hypothesized serial mediational model

Based on previous evidence, this study aimed to examine the associations between female genital self-image, shame, emotional dysregulation, and psychological distress in a sample of women. Specifically, this study had the objective of determining the extent to which female genital self-image, shame, and emotional dysregulation contribute to psychological distress and to test a serial mediation model in which shame and emotional dysregulation sequentially mediate the association between genital self-image and psychological distress. The following hypotheses were formulated (Fig. 1):

- Hypothesis 1 (H1): Higher female genital self-image will be negatively associated with shame, emotional dysregulation, and psychological distress. Shame will be positively associated with emotional dysregulation and psychological distress. Emotional dysregulation will be positively associated with psychological distress.
- Hypothesis 2 (H2): Shame will mediate the direct association between female genital self-image and psychological distress. Specifically, lower levels of female genital self-image will be associated with higher levels of shame, which, in turn, will be linked to greater psychological distress.
- Hypothesis 3 (H3): Emotional dysregulation will mediate the direct association between female genital self-image and psychological distress. Specifically, lower levels of female genital self-image will be associated with greater difficulties in emotion regulation, which, in turn, will be linked to higher psychological distress.
- Hypothesis 4 (H4): Shame and emotional dysregulation will act as serial mediators in the relationship between female genital self-image and psychological distress. Lower levels of female genital self-image will be associated with higher psychological distress through increased shame, which will then contribute to greater emotional dysregulation.

## Methods

### Participants and procedure

This study employed a cross-sectional design. Data was collected through an online survey. The inclusion criteria required participants to be female, at least 16 years old, and proficient in Spanish to understand the questionnaire. The decision to include individuals from the age of 16 was based on the fact that, in the country where the study was conducted, this is the legal age of medical emancipation.

The survey was distributed via a web link, which included an information sheet, an informed consent form, and the assessment questionnaires. The link was shared through social media platforms to reach potential participants. Participation was voluntary, and those who agreed to participate provided their consent before accessing the survey. The study was approved by the Ethics Committee of the University of the first and the last authors and was conducted following ethical guidelines for research involving human participants.

The participants in the final sample were 445 females with a mean age of 40.22 ± 10.69 years (ranging from 17–70). The majority of participants were heterosexual (*n* = 403), held Spanish nationality (*n* = 377), resided in Spain (*n* = 392), and had a university-level education (*n* = 170).

### Measures

In addition to an ad hoc questionnaire with socio-demographic data, the following instruments were used.

The Female Genital Self-Image Scale (FGSIS; Herbenick and Reece 2010) was used to assess female genital self-image. This instrument consists of 7 items (e.g., ‘I feel positively about my genitals’) and a single factor. Participants responded using a 4-point Likert scale, ranging from 1 (strongly disagree) to 4 (strongly agree), with total scores ranging from 7 to 28, where higher scores indicate a more positive genital self-image. In this study, FGSIS showed acceptable internal consistency (α = 0.95). 

The Depression, Anxiety, and Stress Scale-21 (DASS-21; Lovibond and Lovibond 1995; Spanish version: Daza et al. 2002) was used to assess psychological distress. This instrument consists of 21 items (e.g., ‘I felt that life was meaningless’) and comprises three subscales: depression, anxiety, and stress. In this study, the total DASS-21 score was used as a measure of overall psychological distress (Crawford and Henry 2003). Participants responded using a 4-point Likert scale ranging from 0 (did not apply to me at all) to 3 (applied to me very much, or most of the time), with total scores ranging from 0 to 84, where higher scores indicate greater psychological distress. The Spanish version of the DASS-21 demonstrated excellent internal consistency in this sample (α = 0.95).

The SHAME scale (Scheel et al. 2013) was used to assess shame proneness. This instrument consists of 21 items, each representing a specific scenario. It comprises three subscales: two reflecting adaptive aspects of shame—bodily shame (7 items; e.g., “I try on an article of clothing that I can barely fit into. During this, my boyfriend or girlfriend looks into the changing room.”) and cognitive shame (7 items; e.g., “I am praised for something that I did not accomplish myself.”)—and one representing a maladaptive aspect, existential shame (7 items; e.g., “I ask myself what someone else, born instead of me into the same situation, would have done with their life.”). All items are rated on a 6-point Likert scale (0 = “Not at all” to 5 = “An extreme amount”). This scale was translated from English (Scheel et al.) into Spanish by the first and last author. In this study, the SHAME scale demonstrated acceptable levels of internal consistency across all subscales (Bodily: α = 0.80; Cognitive: α = 0.76; Existential: α = 0.78).

The Difficulties in Emotion Regulation Scale (DERS, Gratz and Roemer 2004; Spanish version: Gómez-Simón et al. 2014) was used to assess emotion dysregulation. This scale consists of 36 items focusing on different difficulties of emotional regulation process: Impulse Control Difficulties (Impulse, 6 items, e.g., ‘When I’m upset, I lose control over my behaviors’), Difficulties Engaging in Goal-Directed Behavior (Goals, 5 items, e.g., ‘When I’m upset, I have difficulty focusing on other things’), Lack of Emotional Awareness (Non-awareness, 5 items, ‘I pay attention to how I feel’), Lack of Emotional Clarity (Non-clarity, 5 items, e.g., ‘I am confused about how I feel’), Non-acceptance of Emotional Responses (Non-acceptance, 6 items, e.g., ‘When I’m upset, I feel guilty for feeling that way’), and Limited Access to Emotion Regulation Strategies (Low strategies, 9 items., e.g., ‘When I’m upset, it takes me a long time to feel better’). All items can be answered on a 5-point Likert scale (1 = “Almost never/0–10% of the time”/5 = “Almost always/90–100% of the time”). Higher scores indicate a greater tendency to each difficulty. In this study, the DERS showed acceptable levels of internal consistency in all its subscales (Impulse: α = 0.90; Goals: α = 0.86; Non-awareness: α = 0.81; Non-clarity: α = 0.84; Non-acceptance: α = 0.91; Low strategies: α = 0.88).

### Data analysis

Regarding the distribution of variables, absolute skewness values ranged from 0.039 to 1.331, while absolute kurtosis values varied from 0.067 to 2.452. Following Kim's (2013) guidelines for medium-sized samples (skewness and kurtosis < 3.29), the distribution of all variables was considered approximately normal. Descriptive statistics (means and standard deviations), reliability coefficients (Cronbach's alpha and McDonald's omega), and Pearson correlations among study variables were initially computed.

To examine the specific factors of shame and emotion dysregulation associated with psychological distress, a multiple linear regression analysis was conducted using the enter method, with psychological distress as the outcome variable. The regression model included age, FGSI, shame-related factors (i.e., bodily, cognitive, and existential shame), and emotion dysregulation dimensions (i.e., impulse control difficulties, goal-directed behaviour difficulties, lack of emotional awareness, lack of emotional clarity, non-acceptance of emotions, and limited emotion regulation strategies). Assumptions of linear regression—including normality of residuals, linearity, independence of residuals (DWT = 1.70), homoscedasticity (random scatterplot between residuals and predicted values), and absence of multicollinearity (Tolerance = 0.26–0.84; VIF = 1.21–3.79)—were tested and met. However, due to the violation of the normality assumption of residuals, bootstrapping with 5,000 resamples was applied to estimate B and SE coefficients, ensuring bias-corrected 95% confidence intervals.

Descriptive, correlation, and regression analyses were conducted using JASP statistical software (JASP Team 2025).

To test the hypothesized serial mediation model, we employed the PROCESS macro for SPSS (Version 4.2; Hayes, 2022; IBM Corp. 2021), specifically Model 6, which allows for two serial mediators. FGSI was entered as the independent variable (X), and psychological distress as the dependent variable (Y). Mediators were selected based on their significant association with psychological distress in the previous regression analyses, with shame as the first mediator (M1) and emotion dysregulation as the second mediator (M2). Age was included as a covariate. The mediation analysis was conducted using ordinary least squares (OLS) regression with 5,000 bootstrap resamples to estimate indirect effects and compute bias-corrected 95% confidence intervals (CIs). The statistical significance of the indirect effects was determined by assessing whether the CIs excluded zero.

The minimum required sample size was determined using the G*Power calculator (Faul et al. 2007). Assuming an anticipated effect size of 0.15, a significance level of 0.05, a statistical power of 0.80, and eleven predictor variables, the software estimated a required minimum sample size of 123, which was exceeded in the present study.

## Results

Table 1 presents the Pearson correlations between the study variables. All correlations were statistically significant. While most variables were positively associated, genital self-image showed negative correlations with psychological distress, the three shame dimensions, and the six emotional dysregulation dimensions.

**Table 1:** Pearson correlations between the study variables

	1	2	3	4	5	6	7	8	9	10
1. Psychological distress	—									
2. FGSI	−0.33***	—								
3. Bodily shame	0.38***	−0.48***	—							
4. Cognitive shame	0.30***	−0.27***	0.53***	—						
5. Existential shame	0.45***	−0.37***	0.56***	0.38***	—					
6. Non-awareness	0.30***	−0.33***	0.29***	0.18***	0.30***	—				
7. Impulse	0.54***	−0.30***	0.33***	0.32***	0.32***	0.38***	—			
8. Non-acceptance	0.51***	−0.39***	0.40***	0.40***	0.36***	0.37***	0.59***	—		
9. Goals	0.43***	−0.26***	0.38***	0.29***	0.21***	0.19***	0.62***	0.47***	—	
10. Non-clarity	0.44***	−0.35***	0.35***	0.27***	0.32***	0.66***	0.52***	0.49***	0.40***	—
11. Low strategies	0.64***	−0.45***	0.41***	0.37***	0.38***	0.42***	0.78***	0.69***	0.61***	0.56***

FGSI: Female genital self-image. *** *p* <.001

A multiple linear regression analysis was conducted to examine the associations between FGSI, shame, emotional dysregulation variables, and psychological distress. The model explained 47.0% of the variance (*R²* = 0.47) and was statistically significant (*F*(11, 432) = 34.88, *p* <.001). Existential shame (*β* = 0.246, *p* <.001) and low strategies (*β* = 0.380, *p* <.001) were significantly associated with psychological distress. The remaining variables, including age, FGSI, bodily shame, cognitive shame, non-awareness, impulse, non-acceptance, goals, and non-clarity, did not show significant associations with psychological distress (*p* >.05).

**Fig. 2:**
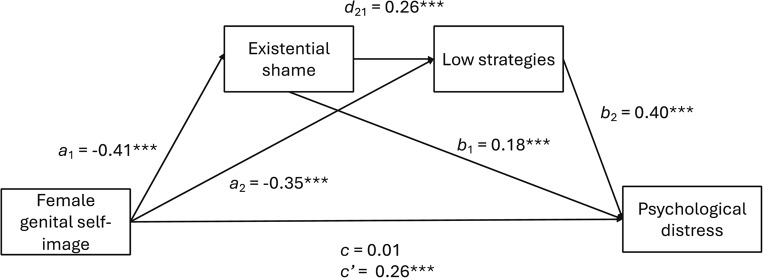
Serial mediation model. ***p <.001

Then, a serial mediation analysis was conducted to examine whether existential shame (M1) and emotional dysregulation (M2) mediate the association between FGSI (X) and psychological distress (Y) while controlling for age (see Table 2; Fig. 2).

**Table 2:** Serial mediation model

Direct associations	B (SE)	*p*	95% CI
FGSI → existential shame	-0.41 (0.05)	<.001	[-0.51, -0.32]
FGSI → low strategies	-0.35 (0.05)	<.001	[-0.43, -0.26]
Existential shame → low strategies	0.26 (0.04)	<.001	[0.18, 0.35]
FGSI → psychological distress	0.01 (0.03)	.819	[-0.06, 0.07]
Existential shame → psychological distress	0.18 (0.03)	<.001	[0.13, 0.24]
Low strategies → psychological distress	0.40 (0.03)	<.001	[0.34, 0.46]
Indirect associations	Effect (BootSE)		95% CI
FGSI → Existential shame → psychological distress	-0.08 (0.02)		[-0.11, -0.05]
FGSI → low strategies → psychological distress	-0.14 (0.02)		[-0.18, -0.10]
FGSI → Existential shame → low strategies → psychological distress	-0.04 (0.01)		[-0.06, -0.03]
Total indirect associations	-0.26 (0.03)		[-0.32, -0.21]

FGSI: Female genital self-image

The model predicting existential shame was significant (*R²* = 0.152, *F*(2, 441) = 39.77, *p* <.001). FGSI was negatively associated with existential shame, indicating that lower FGSI is linked to higher existential shame. The model predicting emotional dysregulation was significant (*R²* = 0.283, *F*(3, 440) = 57.77, *p* <.001). FGSI was negatively associated with low strategies, and existential shame was positively associated with low strategies.

The model predicting psychological distress was significant (*R²* = 0.457, *F*(4, 439) = 92.21, *p* <.001). Existential shame and low strategies were both positively associated with psychological distress, suggesting that higher levels of these variables contribute to increased psychological distress. FGSI did not show a direct association with psychological distress.

Regarding indirect effects, bootstrapped confidence intervals (5000 samples, 95% CI) indicated significant indirect effects of FGSI on psychological distress through existential shame, low strategies, and the serial pathway existential shame → low strategies. The total indirect effect was also significant, supporting a full mediation model where FGSI was associated with psychological distress indirectly through existential shame and low strategies.

## Discussion

This study aimed to examine the relationships between female genital self-image (FGSI), shame, emotion dysregulation, and psychological distress in a sample of women. Specifically, it sought to determine the extent to which FGSI, shame, and emotion dysregulation contribute to psychological distress and to test a serial mediation model in which shame and emotion dysregulation sequentially mediate the association between FGSI and psychological distress. The linear regression model revealed that existential shame and limited access to emotion regulation strategies were significantly associated with psychological distress, whereas FGSI and the remaining shame- and emotion regulation-related variables showed no significant associations with psychological distress. Furthermore, the serial mediation model confirmed that the relationship between FGSI and psychological distress was fully mediated by existential shame, limited access to emotion regulation strategies, and the sequential relationship between these two factors.

Considering the preliminary results of the linear regression model, the finding that existential shame is associated with psychological distress is consistent with previous research indicating that shame is a significant risk factor for psychopathology, both in the general population (Callow et al. 2021; DeCou et al. 2021; López-Castro et al. 2019; Velotti et al. 2017) and in specific populations (Carvalho et al. 2022; LeBlanc et al. 2019). In contrast, no direct association was found between bodily and cognitive shame and psychological distress. This finding is in line with the conceptual framework of the assessment instrument, which defines existential shame as the maladaptive dimension of shame, whereas bodily and cognitive shame are considered adaptive dimensions (Scheel et al. 2020) and, therefore, would not necessarily be expected to be linked to psychological distress. Additionally, the absence of a direct association between bodily and cognitive shame and psychological distress may be explained by the mediating role of existential shame. That is, increased bodily and cognitive shame may contribute to heightened psychological distress through an increase in existential shame. Indeed, previous research has highlighted cultural differences in the relationship between these three facets of shame and psychopathology (Scheel et al. 2020).

Similarly, the association between limited access to emotion regulation strategies and psychological distress is consistent with the notion that emotion dysregulation is a common risk factor for psychopathology (Aldao et al. 2016). The fact that only this specific subscale of DERS was significantly associated with psychological distress may be due to the greater impact of having limited access to effective emotion regulation strategies in predicting psychological distress. Previous research has reported stronger correlations between this subscale and the three subscales of the DASS-21 compared to other DERS factors (Ma and Fang 2019).

On the other hand, the total association between FGSI and psychological distress was fully mediated by existential shame and limited access to emotion regulation strategies. First, existential shame mediated this relationship, providing further evidence that women with a negative FGSI may experience heightened existential shame due to not meeting socially constructed ideals of female genital image, which, in turn, is associated with greater psychological distress (McDougall 2013; Michala 2020).

Second, the association between FGSI and psychological distress was mediated by limited access to emotion regulation strategies. This suggests that women with a negative FGSI and restricted access to effective regulatory strategies may experience higher levels of psychological distress. This finding can be explained by the negative impact of FGSI on women’s well-being, particularly in areas such as sexual functioning (Alavi-Arjas et al. 2023), which, when combined with a lack of effective emotion regulation strategies, may contribute to increased emotional distress. This aligns with previous research demonstrating that difficulties in emotion regulation mediate the relationship between body image dimensions and psychopathology (Duggan et al. 2013).

Furthermore, the indirect effect of FGSI on psychological distress through the sequential mediation of existential shame and limited access to emotion regulation strategies suggests a cumulative mechanism. Women with a negative FGSI may experience heightened shame (Kvalem et al. 2024), which, when paired with difficulties in regulating emotions, could lead to greater psychological distress. Moreover, emotional dysregulation has been shown to mediate the link between shame and psychological distress (Remondi et al. 2023; Velotti et al. 2017), further supporting the notion that difficulties in regulating emotions exacerbate the impact of shame on mental health outcomes.

This study is not without limitations. First, its cross-sectional design prevents the determination of causal relationships between the study variables. To address this limitation, future research should replicate these findings using longitudinal designs to clarify the directionality of these associations. Additionally, subsequent studies could employ experimental interventions to investigate causal pathways. For example, implementing an intervention aimed at negative female genital self-image, existential shame, and difficulties in emotion regulation, followed by assessing subsequent changes in psychological distress, could prove beneficial. Second, the study relied exclusively on self-report measures, which are subject to limitations such as social desirability bias and recall bias. To gain a more comprehensive understanding of the studied phenomena, future research should incorporate complementary assessment methods, such as behavioural, cognitive, or emotional records, as well as interviews. Additionally, it is recommended to use the *Process Model of Emotion Regulation Questionnaire* (PMERQ; Olderbak et al. 2022) to assess emotion regulation, as this would help identify the specific stage of the emotion regulation process where difficulties related to FGSI, shame, and psychological distress arise in women. Third, the sample consisted almost entirely of Spanish women residing in Spain, which may limit the generalisability of the findings to populations from different sociocultural backgrounds. Future research should investigate the extent to which cultural differences, particularly in societal attitudes toward sexuality and gender roles, may affect the interrelations among female genital self-image, shame, emotion regulation, and psychological distress. Fourth, and also related to the generalisability of the results, data collection was conducted via online surveys distributed through social media, which restricted access to women who were not digitally connected. To address this limitation, future studies should complement online data collection with in-person surveys to ensure a more representative sample.

Despite these limitations, this study makes significant contributions to the field. It is the first to date to explore the relationships between FGSI, shame, and emotion dysregulation, as well as to test a serial mediation model explaining the association between FGSI and psychological distress through shame and emotion dysregulation. The findings have important practical implications for women’s well-being, highlighting the need for interventions targeting women with a negative genital self-image. Such interventions should focus on addressing feelings of shame and improving emotion regulation, with particular emphasis on enhancing access to effective strategies for managing shame-related negative emotions.

Various evidence-based therapeutic approaches have been identified as potentially beneficial. Acceptance and Commitment Therapy has been shown to be effective in transforming negative shame and enhancing emotion regulation (Khoramnia et al. 2020; Lear and Luoma 2025; Yuan et al. 2024). Additionally, Cognitive Behavioral Therapy, Dialectical Behavior Therapy, and Mindfulness-Based Interventions have demonstrated efficacy in improving emotion regulation (Antuña-Camblor et al. 2024; Guendelman et al. 2017; Sloan et al. 2017). Furthermore, Emotion Regulation Therapy and experiential therapies specifically target self-criticism and shame (Iwakabe et al. 2023; Renna et al. 2020). These therapeutic approaches provide a robust foundation for the development of targeted interventions aimed at addressing the core mechanisms identified in this study.

## Conclusions

This research provides strong evidence of a significant relationship between female genital self-image, existential shame, and limited access to emotion regulation strategies. Specifically, the findings suggest that female genital self-image is indirectly linked to psychological distress through two key mediating mechanisms: existential shame and emotional dysregulation. The serial mediation pathway indicates that a more negative female genital self-image is associated with heightened existential shame, which, in turn, contributes to greater difficulties in emotional regulation, ultimately leading to increased psychological distress. These results underscore the critical role of self-perception, shame proneness, and emotional regulation in women’s psychological well-being, highlighting the need for interventions that address these interconnected factors to promote mental health.

## Data Availability

Data available on request due to privacy/ethical restrictions.
